# Contribution of Particle-Induced Lysosomal Membrane Hyperpolarization to Lysosomal Membrane Permeabilization

**DOI:** 10.3390/ijms22052277

**Published:** 2021-02-25

**Authors:** Tahereh Ziglari, Zifan Wang, Andrij Holian

**Affiliations:** 1 Department of Biomedical and Pharmaceutical Sciences, Center for Environmental Health Sciences, University of Montana, Missoula, MT 59812, USA; tahereh.ziglari@umontana.edu; 2 Division of Chemistry and Biochemistry, College of Humanities and Sciences, University of Montana, Missoula, MT 59812, USA; zifan.wang@mso.umt.edu

**Keywords:** lysosomal membrane potential, nanoparticles, zinc oxide nanoparticles, crystalline silica

## Abstract

Lysosomal membrane permeabilization (LMP) has been proposed to precede nanoparticle-induced macrophage injury and NLRP3 inflammasome activation; however, the underlying mechanism(s) of LMP is unknown. We propose that nanoparticle-induced lysosomal hyperpolarization triggers LMP. In this study, a rapid non-invasive method was used to measure changes in lysosomal membrane potential of murine alveolar macrophages (AM) in response to a series of nanoparticles (ZnO, TiO_2_, and CeO_2_). Crystalline SiO_2_ (micron-sized) was used as a positive control. Changes in cytosolic potassium were measured using Asante potassium green 2. The results demonstrated that ZnO or SiO_2_ hyperpolarized the lysosomal membrane and decreased cytosolic potassium, suggesting increased lysosome permeability to potassium. Time-course experiments revealed that lysosomal hyperpolarization was an early event leading to LMP, NLRP3 activation, and cell death. In contrast, TiO_2_- or valinomycin-treated AM did not cause LMP unless high doses led to lysosomal hyperpolarization. Neither lysosomal hyperpolarization nor LMP was observed in CeO_2_-treated AM. These results suggested that a threshold of lysosomal membrane potential must be exceeded to cause LMP. Furthermore, inhibition of lysosomal hyperpolarization with Bafilomycin A1 blocked LMP and NLRP3 activation, suggesting a causal relation between lysosomal hyperpolarization and LMP.

## 1. Introduction

Development of nanotechnology in recent years has resulted in rapid expansion in production and utilization of nanoparticles (NP) [1]. NP are created materials with particle sizes between 1 to 100 nanometers in at least one dimension [2]. Zinc oxide (ZnO), titanium dioxide (TiO_2_), and cerium oxide (CeO_2_) nanoparticles have been frequently used in consumer products [3], consequently, their widespread use increases the likelihood that these materials will result in human exposures [4]. Many studies have reported that some NP have the potential to cause toxicity and inflammation [5,6,7,8,9] following NRLP3 inflammasome activation [10,11,12]. Alveolar macrophages (AM) are the key innate immune cells in lungs responsible for the recognition and removal of inhaled particles that can lead to NLRP3 inflammasome activtion [5,11]. However, the mechanisms accounting for how NP or common micron-sized particles such as respirable crystalline silica (SiO_2_, which is known to cause silicosis) activate the NLRP3 inflammasome remain unclear.

Depletion of cytosolic potassium (K^+^) has emerged as a common denominator in the activation of the NLRP3 inflammasome [13,14,15]. Early studies demonstrated that K^+^ ionophores such as nigericin could trigger IL-1β maturation in macrophages [16]. Additional studies suggested that cytosolic K^+^ depletion by nigericin alone is a minimal common cellular event that is sufficient to activate the NLRP3 inflammasome [14,17]. Other studies have proposed a role for particle-induced lysosomal membrane permeability (LMP) in inflammasome activation and cell death [11,18,19]. However, the exact underlying mechanisms leading to LMP have not been described and determination of whether there is any relationship between LMP and changes in cytosolic [K^+^] remain to be clarified.

One possible mechanism underlying LMP may involve an induced osmotic imbalance across the lysosomal membrane [20,21,22]. There is an inherent association between osmotic balance across the lysosomal membrane and lysosomal membrane potential [23], that contributes to lysosomal integrity [22]. Lysosomal membrane potential is determined by ionic permeability and is modulated by ion channels and changes in intraluminal versus extraluminal ionic concentrations [23]. To date, efforts to measure lysosomal membrane potential have been challenging due to inefficient methods for measuring electrophysiology in cellular organelles, as well as a lack of specific potentiometric dyes for lysosomes. Furthermore, the use of potentiometric fluorescent dye intensity as an indicator of lysosomal membrane potential is hampered by a dependence on knowing the concentration of potentiometric dyes in the cytosol. Koivusalo et al. (2011) devised a novel technique using fluorescence resonance energy transfer (FRET) to measure lysosomal membrane potential, which relies on a calibration curve created by various treatments to simultaneously dissipate plasma and lysosomal membrane potentials [24]. However, FRET has some drawbacks such as having low signal-to-noise ratio and being time-consuming. Furthermore, the treatments for creating a calibration curve reduce cell viability of sensitive cell populations.

The goal of the current study was to address the hypothesis that NP and SiO_2_ can induce lysosomal membrane hyperpolarization following an increase in lysosomal membrane permeability to cations (predicted to be K^+^), which could lead to a progressive osmotic imbalance across the lysosomal membrane and LMP. This study proposes a correlation between cytosolic K^+^ decrease and LMP in NP-induced inflammatory responses. AM isolated from C57B1/6 mice and two NP that are currently being produced in high amounts, ZnO, and TiO_2_ [25] were used to determine their predicted contrasting effects on lysosomal membrane potential. Micron-sized SiO_2_ was used as a positive control based on its well-defined toxicity profile [26]. CeO_2_ was used as a test particle based on a variety of reports including anti-oxidant characteristics [2]. In order to facilitate measurements of lysosomal membrane potential, a rapid non-invasive method was developed to directly measure lysosomal membrane potential based on the cytosolic-lysosomal ratio (C_C_/C_L_) of the fluorescent intensity of DiBAC4(3). Using the C_C_/C_L_ ratio of the dye excludes the effect of the variances of the cytosolic concentration of the dye and possible distribution of DiBAC4(3) to other organelles on its lysosomal concentration.

## 2. Results

### 2.1. Characterization and Cytotoxicity of NP and SiO_2_

**Figure 1:**
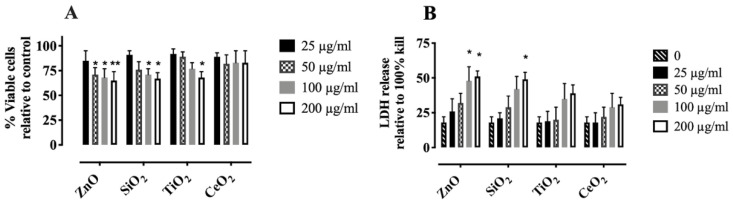
Toxicity in AMattributable to NP and SiO_2_. AM were incubated with individual particles for 4 h at 37 °C. Results from the (**A**) MTS assay after incubation of particles with AM, (**B**) LDH assay. Data are presented as means ± SEM of triplicate measurements. * and ** indicates significant effects (*p* ≤ 0.05 and *p* ≤ 0.01, respectively). Data analyzed by two-way ANOVA using Holm–Sidak’s post hot test.

The NP and SiO_2_ used in this study have been previously characterized [5,27,28,29]. Consistent with those descriptions, transmission electron microscopy (TEM) images showed NP and SiO_2_ to be approximately 30 nm and 1 µm in diameter, respectively (Table S1, Figure S1). To determine the hydrodynamic size, the materials were suspended in RPMI media containing 10% FBS and zeta-potential measurements of the NP were made using a ZetaSizer Nano-ZS (Malvern Instruments, Worcestershire WR, UK). In order to confirm the relative toxicity of the materials, cellular viability of AM was determined using standard MTS and LDH assays after 1, 2, and 4 h incubation with SiO_2_ or NP (0–200 µg/mL) and resultant downstream NLRP3 inflammasome activity (extracellular IL-1β levels) after 24 h. As expected, a dose-dependent decrease in cell viability of freshly isolated AM (from C57B1/6) to ZnO or SiO_2_ was observed, but not earlier than 4 h (Figure 1 and Figure S2). In contrast, the same concentrations and incubation conditions of TiO_2_ or CeO_2_ did not cause significant toxicity in AM incubated at the same experimental conditions, except for a high 200 µg/mL dose of TiO_2_ at in 4 h (Figure 1).

### 2.2. Validation of Lysosomal Membrane Hyperpolarization Using DiBAC4(3)

The anionic potentiometric probe DiBAC4(3) has been previously used to measure plasma membrane potential [30,31,32]. In this study, DiBAC4(3) was used to develop a rapid non-invasive method for measuring lysosomal membrane. Since DiBAC4(3) is soluble and membrane-permeant, it distributes throughout cells, but excluded from mitochondria due to their internal negative charge [33]. The results showed that DiBAC4(3) accumulated inside lysosomes (Figure S3) in response to their internal positive charge [23]. Therefore, changes in the lysosomal membrane potential, attributable to variations in lysosomal ionic concentrations, would be expected to affect DiBAC4(3) concentration inside lysosomes and could be measured using confocal microscopy.

**Figure 2:**
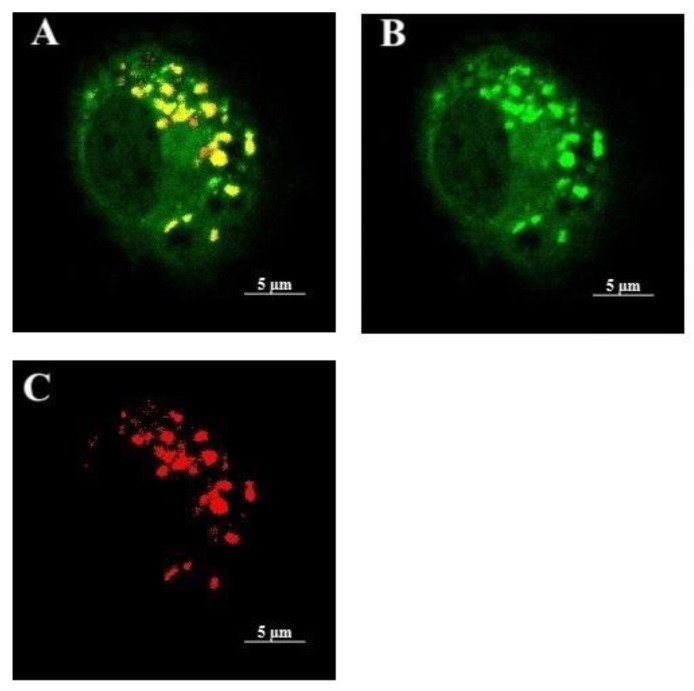
Distribution of DiBAC4(3) (Ex/Em 493/516) and Lysotracker Red (Ex/Em 577/590) in live AM. (**A**) Overlay, (**B**) DiBAC4(3), and (**C**) Lysotracker Red. The lysosomes were labeled simultaneously with 300 nM DiBAC4(3) and 50 nM Lysotracker Red. The colocalization relative to Lysotracker was measured using a Zeiss LSM 880 confocal microscope. The concentration of dyes was shown to be optimal after 1 h incubation.

In order to selectively distinguish the membrane potential changes assigned to lysosomes, the following validations were performed. First, the relatively selective presence of DiBAC4(3) in lysosomes was demonstrated by simultaneously treating AM with DiBAC4(3) and Lysotracker Red, a probe with high selectivity for acidic organelles [28]. Overlap of DiBAC4(3) and Lysotracker Red in the lysosomes was qualitatively visualized via confocal microscopy in most cells (Figure 2). Furthermore, co-localization analysis of about 100 cells using ImageJ quantitatively demonstrated a high overlap of the two dyes with a Pearson correlation coefficient of 0.87 ± 0.08. Although DiBAC4(3) distribution is not entirely lysosome-specific, the high overlap of DiBAC4(3) and Lysotracker Red clearly showed that DiBAC4(3) predominantly accumulated inside lysosomes within AM.

Second, the pH-independency of DiBAC4(3) quantum yield was determined. The spectra of DiBAC4(3) fluorescent intensity at different pH indicated that the quantum yield of the dye was not pH-dependent (Figure S4). Furthermore, a calibration curve of increasing DiBAC4(3) concentrations showed that lysosomal fluorescence of DiBAC4(3) was linearly correlated with its concentration (Figure S5).

Third, the cytosolic to lysosomal, C_C_/C_L_, ratio of the fluorescent intensity of DiBAC4(3) in over 100 cells was calculated using ImageJ within individual cells. For this purpose, AM were incubated in RPMI media containing 10% FBS. The media was then replaced with PBS containing 300 nM DiBAC4(3) for 10 min as described in the Methods. As previously described by Koivusalo et al. (2011) [24], the lysosomal concentration of DiBAC4(3) is dependent on both cytosolic concentration of the dye and lysosomal membrane potential, while the cytosolic concentration of the dye is a function of plasma membrane potential. Using the C_C_/C_L_ ratio of the dye allows the effect of the variances of cytosolic concentration and possible distribution of the dye to other organelles to be excluded. Therefore, the calculated lysosomal membrane potentials are most likely specifically assigned to lysosomes. Next, the C_C_/C_L_ ratio of DiBAC4(3) was applied using the Nernst equation as described in the Methods to calculate lysosomal membrane potential (Table S2). Lysosomes at rest were estimated to have a lumen-positive value of 23.8 ± 2.8 mV, which is similar to previous reports of lysosomal membrane potential measured by FRET (19 mV) in RAW264.7 macrophages [24] or by current-clamp recording (30 mV) in isolated lysosomes [34].

Fourth, the sensitivity of this assay for detecting measurable changes in lysosomal membrane potential was examined. For this purpose, lysosomal membrane potential was manipulated with either Valinomycin, a specific K^+^ ionophore, or Bafilomycin A1, a selective inhibitor of vacuolar H^+^-ATPases responsible for lysosome acidification, as discussed in the Methods [28]. Valinomycin is a specific K^+^ inophore, which incorporates into both plasma membrane and lysosomal membranes [34,35] increasing K^+^ permeability across the lysosomal membrane [22] and facilitating K^+^ efflux from cytosol to extracellular fluid [36]. Therefore, valinomycin was used as an inducer of lysosomal hyperpolarization. The increased K^+^ efflux hyperpolarizes the lysosomal membrane [23]. Hyperpolarization of the lysosomal membrane of AM after treating with different doses of Valinomycin (100 nm, 2 µM, and 20 µM) was detected using this assay (Figure S6A). Wang et al. showed that removal of lysosomal H^+^ resulted in an increase in lysosomal pH and decreased lysosomal membrane potential [34]. Therefore, in this study, Bafilomycin A1 was used as a positive control of lysosomal membrane potential reduction due to the effect of H^+^ in increasing lysosomal membrane potential [23]. A reduction of lysosomal membrane potential (~12 mV) was observed in Bafilomycin A1-treated AM compared to control (~23 mV) (Figure S6A) following a decrease in lysosomal H^+^ (Figure S6B). Together, these results suggested that the assay established here could accurately measure the lysosomal membrane potential and is sensitive to detect alterations in lysosomal membrane potential.

Finally, in order to demonstrate that the various particles do not interfere with DiBAC4(3) or the fluorescent signal generated, DiBAC4(3) was loaded into 100 µm-liposome vesicles as described in Methods. The emission fluorescent spectra of liposomes with and without NP were measured with spectrophotometry. Liposomes containing DiBAC4(3) and its quencher, L-∝-phosphatidylethanolamine-N-lissamine rhodamine B sulfonyl (Rh-PE) was used as a control for fluorescent intensity reduction of DiBAC4(3). The areas under the curve of the spectra (liposome with and without NP) were not significantly different, suggesting that NP do not interfere with the fluorescent intensity of the dye (Figure S7).

### 2.3. Effects of NP and SiO_2_ on Lysosomal Membrane Potential

**Figure 3:**
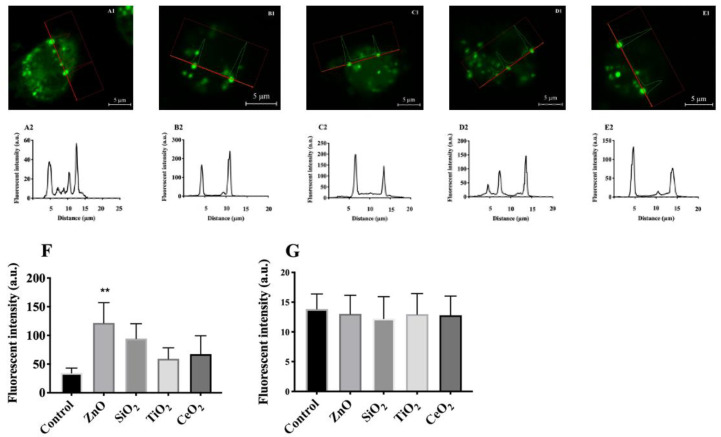
Comparison of the distribution of DiBAC4(3) in control vs particle-treated AM. Confocal images (A1-E1) and representative examples of fluorescent intensity (A2-E2) of DiBAC4(3) in the lysosomes of live AM. AM were incubated with individual particles for 1 h at 37 °C. (**A1**,**A2**) control, (**B1**,**B2**) treated with 100 µg/mL ZnO, (**C1**,**C2**) treated with 100 µg/mL SiO_2_, (**D1**,**D2**) treated with 100 µg/mL TiO_2_ and (**E1**,**E2**) treated with 100 µg/mL CeO_2_. (**F**) Distribution of DiBAC4(3) in the lysosome of control vs treated cells analyzed by one-way ANOVA. (**G**) Distribution of DiBAC4(3) in the cytosol of control vs treated cells analyzed by one-way ANOVA. The fluorescence intensity of at least 100 cells per group was measured and analyzed using ZEN Black imaging software (ZEISS) and ImageJ. Data are presented as means ± SEM of triplicate measurements. ** Indicates significant effect (*p* ≤ 0.01) by Holm–Sidak’s multiple comparison test.

The effects of particles on lysosomal membrane potential was determined using AM incubated with the three NP or SiO_2_ (0–200 µg/mL) for one hour in RPMI media containing 10% FBS. The media was then replaced with PBS containing 300 nM DiBAC4(3) for 10 min as described in the Methods. Since none of the particles caused LMP within an hour (Figure S8), this time point was selected to test the hypothesis that particles could cause lysosomal hyperpolarization and would be upstream of LMP. To determine particle-induced changes in lysosomal membrane potential, the C_C_/C_L_ ratio of DiBAC4(3) of over 100 cells in each group was first calculated by ImageJ as described in the Methods. The results indicated that the ratio was lower in AM treated with ZnO, TiO_2_, CeO_2_, or SiO_2_ than in control cells (Figure 3). The exact ratios were 0.1, 0.223, 0.23, 0.12, and 0.4 in 100 µg/mL ZnO-, TiO_2_-, CeO_2_-, SiO_2_-treated AM, and control, respectively (Table S2). Compared to the control (Figure 3A1,A2), ZnO or SiO_2_ caused the most dramatic effects in AM (Figure 3B1, B2,C1,C2); while negligible effects were observed in AM treated with TiO_2_ or CeO_2_ (Figure 3D1,D2,E1,E2). The lower C_C_/C_L_ ratio of DiBAC4(3) in treated cells was attributable to the shift in increased fluorescent intensity of the dye in the lysosomes of treated cells than in control cells (Figure 3F), while the fluorescent intensity of the dye in the cytosol of control and treated cells remained unchanged (Figure 3G). Consequently, the increase in fluorescent intensity of the anionic dye in the lysosomes was likely the result of lysosomal cation uptake [23,24].

**Figure 4:**
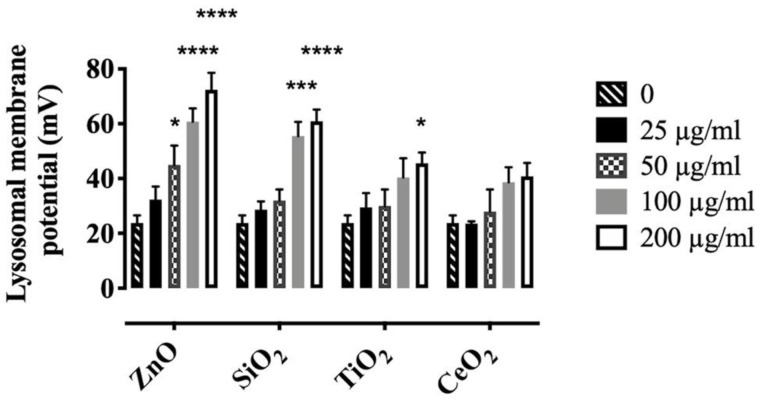
Hyperpolarization of AM lysosomal membranes after exposure to NP or SiO_2_. AM were incubated with particles for 1 h at 37 °C and lysosomal membrane potential was calculated as discussed in the Methods. A Zeiss LSM 880 confocal microscope and ZEN imaging software (ZEISS) as well as ImageJ were used for our studies. Data are presented as means ± SEM of triplicate measurements. *, ***, and **** indicate significant effects (*p* ≤ 0.05, *p* ≤ 0.001, and *p* ≤ 0.0001, respectively). Data analyzed by two-way ANOVA and Holm–Sidak’s post hoc test.

Next, the C_C_/C_L_ ratio of DiBAC4(3) was used to calculate lysosomal membrane potential as described in Methods (Table S2). A significant dose-dependent hyperpolarization of the lysosomal membrane potential was observed in AM treated with ZnO and SiO_2_ compared to control cells. In contrast, TiO_2_ or CeO_2_ caused only modest changes in lysosomal membrane hyperpolarization in AM, except for TiO_2_ at 200 µg/mL. Lysosomal membrane potential changed to 60.8 ± 4.8, 55.54 ± 5.12, 40.4 ± 7.9, and 38.61 ± 5.5 mV when treated with 100 µg/mL ZnO, SiO_2_, TiO_2_, or CeO_2_, respectively (Figure 4). Since lysosomal hyperpolarization would most likely occur due to cation uptake [23], these results provide additional evidence of lysosomal cation uptake attributable to exposure of AM to ZnO or SiO_2_.

### 2.4. Effects of Particles on Cytosolic Potassium [K^+^]

**Figure 5:**
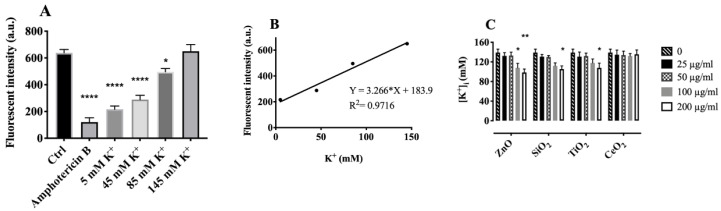
Cytosolic K^+^ depletion attributable to individual NP or SiO_2_ exposure. Cytosolic K^+^ was measured using acetoxymethyl ester of APG-2 as described in Methods. (**A**) AM were depleted of K^+^ and Na^+^ (one way ANOVA) (**B**) Calibration curve as described in Methods. (**C**) Incubation of AM with NP or SiO_2_ for 1 h at 37 °C lead to decreased cytosolic K+ analyzed by two-way ANOVA. Data are presented as means ± SEM of triplicate measurements. *, **, **** indicate significant effects (*p* ≤ 0.05, *p* ≤ 0.01 and *p* ≤ 0.0001 respectively) according to Holm–Sidak’s multiple comparions test.

The above results suggested that the increased membrane hyperpolarization induced by ZnO and SiO_2_ most likely resulted from lysosomal cation uptake, leading to lysosomal membrane hyperpolarization in AM. Under normal conditions, lysosomes are more acidic (pH 4.6) and have much lower levels of [K^+^] ~ 5 mM than the cytosol, which has [K^+^] of approximately 140 mM [34]. Cytosolic [K^+^] was measured using Asante potassium green-2 (APG-2), a K^+^ indicator, as described in Methods. K^+^ sensitivity and selectivity of APG-2 was first evaluated using spectrofluorimetry. The data indicated that APG-2 was sufficiently sensitive to monitor K^+^ and specifically detected K^+^ in the presence of Na^+^ (Figure S9). To determine cytosolic [K^+^] accurately, complete plasma membrane depolarization was required [37]. For this purpose, K^+^-rich buffer and amphotericin B were used to induce depolarization across the plasma membrane (Figure S10). To measure plasma membrane potential, DiBAC4(3) was used in a similar manner as previously described [32]. An in situ calibration procedure (Figure 5A,B) enabled an estimate of the resting cytosolic [K^+^] to be 139 ± 7 mM (Figure 5C), which was consistent with previous reports [37,38]. The addition of either ZnO or SiO_2_ to AM induced a dose-dependent decrease in cytosolic [K^+^], while TiO_2_ or CeO_2_ caused only a negligible decrease in cytosolic [K^+^], except for TiO_2_ at 200 µg/mL, which caused significant cytosolic K^+^ depletion. Based on the calibration curve, the resulting cytosolic K^+^ concentrations following particle treatments were estimated to be 108.56 ± 9.00, 112.43 ± 6.44, 118.89 ± 8.60, and 132.59 ± 4.80 mM in AM treated with 100 µg/mL ZnO, SiO_2_, TiO_2_, and CeO_2_, respectively (Figure 5C). These results were consistent with the lysosomal membrane hyperpolarization results, in which ZnO and SiO_2_ dose-dependently increased lysosomal membrane potential, but not TiO_2_ and CeO_2_, except when the concentration of TiO_2_ reached 200 µg/mL. These results suggest a positive correlation between lysosomal hyperpolarization and cytosolic K^+^ decrease.

In order to determine whether lysosomal membrane hyperpolarization and cytosolic [K^+^] decrease was the result of lysosomal K^+^ influx rather than cytosolic K^+^ efflux to the extracellular compartment, cellular K^+^ efflux was inhibited by suspending AM in a K^+^-rich buffer [39]. The incubation time in K^+^-rich buffer did not exceed 1 h and the viability of AM in K^+^-rich buffer was not significantly different from that of AM in RPMI medium (data not shown). Inhibition of K^+^ efflux in high extracellular K^+^ did not prevent lysosomal hyperpolarization (Figure S11) or cytosolic [K^+^] decrease (Figure S12) by ZnO- or SiO_2_-treatment of AM. These results suggest that lysosomal hyperpolarization and cytosolic K^+^ decrease were likely the result of lysosomal K^+^ influx into the lysosome rather than cytosolic K^+^ efflux to extracellular compartment. However, the lower hyperpolarization (Figure S11) and the higher cytosolic [K^+^] (Figure S12) in AM suspended in K^+^-rich buffer than in AM suspended in phosphate buffered saline (PBS) (Figure 4 and Figure 5C), were likely due to the effects of the changes in cation concentrations in the extracellular compartment.

### 2.5. Effects of Lysosomal H^+^-Influx on Lysosomal Hyperpolarization

**Figure 6:**
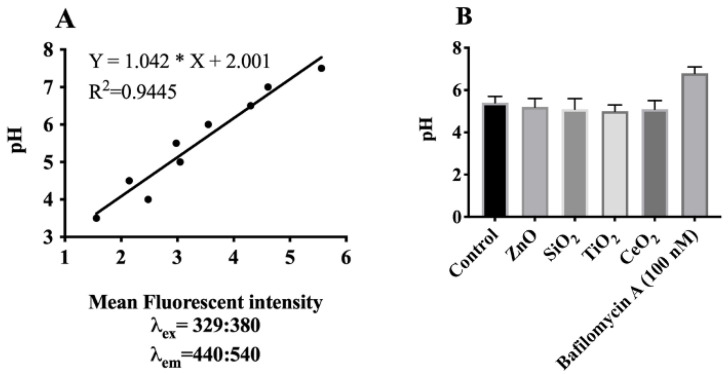
Estimation of intralysosomal pH in AM using ratiometric dye LysoSensor Yellow/Blue DND-160. (**A**) Calibration curve was generated using a SpectraMax M4 spectrofluorometer as described in the Methods. Emission spectra were collected at 440 and 540 nm for excitations at 329 and 380 nm, respectively. (**B**) Fluorescence intensity ratios of individual NP- or SiO_2_-treated cells were converted to pH-values by fitting of the data to the corresponding pH calibration curve. Data are presented as means ± SEM of triplicate measurements. Data were analyzed by two-way ANOVA according to Holm–Sidak’s multiple comparion test.

As described above, our results suggested that the lysosomal cation uptake (lysosomal membrane hyperpolarization) observed in AM treated with ZnO or SiO_2_ was likely attributable to lysosomal influx of K^+^. In order to determine the extent of any contribution of a H^+^ gradient change to NP- or SiO_2_-induced lysosomal hyperpolarization, experiments were conducted to measure phagolysosome pH changes. The intra-lysosomal pH of AM was measured using Lysosensor Yellow/Blue DND-160 [40], providing an in situ estimate of lysosomal pH (Figure 6A,B). The relative mean fluorescent intensity of NP- or SiO_2_-treated cells indicated a negligible decrease in pH (corresponding to a negligible increase in H^+^) of the lysosomes over that of control cells (Figure 6B). These results suggest that in addition to K^+^, H^+^ movement contributed only a minor extent to lysosomal membrane hyperpolarization. Inhibition of vacuolar H^+^-ATPases with bafilomycin A1 simultaneously led to a decrease in lysosomal membrane potential (Figure S6A) and an increase in lysosomal pH as expected (Figure S6B).

### 2.6. Relationship between Lysosomal Hyperpolarization, LMP, and NLRP3 Inflammasome Activity

**Figure 7:**
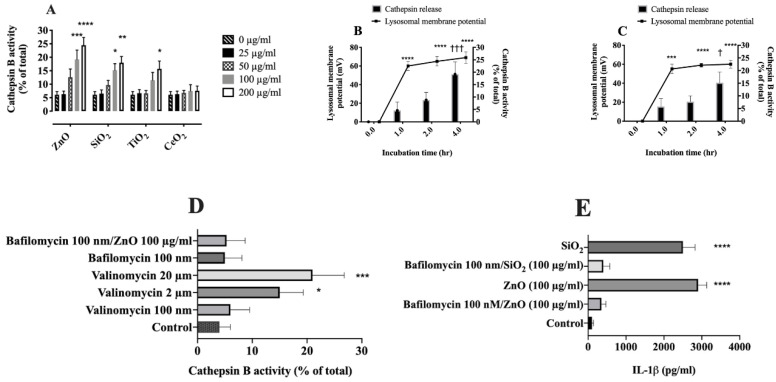
Particle-induced lysosomal hyperpolarization in AM in an early event preceding LMP. (**A**) Increase in cathepsin B activity in AM exposed to three NP or SiO_2_ (0–200 µg/mL) and LMP assessed by two-way ANOVA as described in the Methods. (**B**) LMP and lysosomal hyperpolarization of AM treated with 100 μg/mL of ZnO were measured at 4 time points (0, 1, 2, and 4 h) at 37 ℃ and analyzed by two-way ANOVA. (**C**) LMP and lysosomal hyperpolarization of AM treated with 100 μg/mL of SiO_2_ were measured at 4 time points (0, 1, 2, and 4 h) and analyzed by two-way ANOVA. (**D**) AM were incubated with Valinomycin, Bafilomycin A1, and a cocktail containing Bafilomycin A1 and ZnO (100 µg/mL) for 4 h at 37 ℃ and subsequent cathepsin B release was assessed and analyzed by one-way ANOVA. (**E**) IL-1β levels in supernatants of AM after exposure to three NP or SiO_2_ (100 µg/mL) and analyzed by one-way ANOVA. Data are presented as means ± *SEM* of triplicate measurements. *,**,***, and **** indicate significant effect (*p* ≤ 0.05, *p* ≤ 0.01, *p* ≤ 0.001, and *p* ≤ 0.0001). † and ††† indicates significant effect (*p* ≤ 0.05 and *p* ≤ 0.001) according to Holm–Sidak’s multiple comparison test.

The final question this study addressed was whether NP- or SiO_2_-induced lysosomal hyperpolarization preceded LMP. For this purpose, released cathepsin B (lysosomal hydrolase) was measured in the cytosol as an indicator of LMP [41]. The results indicated that ZnO or SiO_2_ dose-dependently induced cathepsin B release from the lysosome to cytosol within 4 h, while TiO_2_- or CeO_2_-treated AM, cathepsin B release was not significant (Figure 7A), except for TiO_2_ at 200 µg/mL, which first led to lysosomal hyperpolarization (Figure 4). The time-course experiments in AM treated with ZnO or SiO_2_ revealed released cathepsin B in cytosol took approximately 4 h (Figure S8A,B and Figure 7A), while hyperpolarization was detected within 1 h after incubation of AM with ZnO or SiO_2_ (Figure 4 and Figure 7B,C). These data suggest that lysosomal hyperpolarization is an early event preceding LMP.

In order to determine any causative relationship between lysosomal hyperpolarization and LMP, lysosomal hyperpolarization was induced after exposing AM to Valinomycin and measuring impacts on LMP. The results indicated that exposure of AM to Valinomycin (20 or 2 µM) for 1 h dramatically hyperpolarized lysosomes (Figure S6A), which was accompanied by LMP within 4 h (Figure 7D). A lower concentration of Valinomycin (100 nM), which slightly hyperpolarized the lysosomal membrane (Figure S6A) did not cause LMP within 4 h (Figure 7D), suggesting a causal link between lysosomal hyperpolarization and LMP. Previous studies indicated that Valinomycin induced an abrupt cytosolic acidification [42], which eventually leads to cell death [42,43], however, the underlying mechanism of cytosolic acidification remains to be determined.

Furthermore, Bafilomycin A1 was shown to reduce lysosomal membrane potential. A cocktail of Bafilomycin A1 and ZnO (100 µg/mL), which resulted in a lysosomal membrane potential change from ~23 mV (control) to ~32 mV, (Figure S6A) did not lead to LMP in 4 h (Figure 7D); while ZnO (100 µg/mL), which increased lysosomal membrane potential to ~60 mV (Figure 4) caused significant LMP in 4 h (Figure 7A). These results suggest that a certain threshold in lysosomal membrane potential must be exceeded to cause LMP. Consistent with these results, it was previously reported that Bafilomycin A1 inhibited LMP by increasing lysosomal pH [28]. Furthermore, inhibition of lysosomal hyperpolarization and LMP with Bafilomycin A1 also decreased NLRP3 inflammasome activity as measured by the release of IL-1β in ZnO- or SiO_2_-treated AM (Figure 7E).

## 3. Discussion

**Figure 8:**
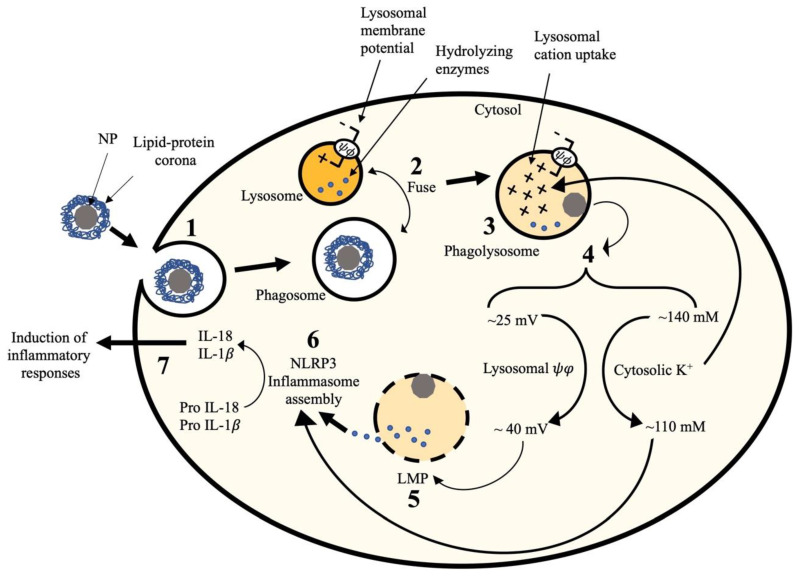
NP-induced lysosomal hyperpolarization triggers LMP. (1) NP covered with lipid-protein corona are internalized into macrophage, creating phagosome. (2) Phagosome ifuses with lysosome creating phagolysosome containing hydrolyzing enzymes in an acidic pH. (3) NP interactn with phagolysosomal membrane. (4) This interaction causes two simultaneous changes in macrophage: (A) decreasing the cytosolic K^+^ and (B) increasing lysosomal membrane permeability to cation, which is likely K^+^ translocating from cytosol to lysosome, leading to lysosomal membrane hyperpolarization. (5) Lysosomal hyperpolarization osmotically disturbs lysosomal integrity, which eventually leads to LMP. (6) The release of lysosomal hydrolyzing enzymes as a result of LMP induces NLRP3 inflammasome activation, including the release of proinflammatory cytokines such as IL-1β. (7) Release of proinflammatory cytokines induces the inflammatory responses.

This study was designed to investigate the underlying mechanism of particle-induced LMP. A rapid non-invasive procedure for measuring lysosomal membrane potential was developed and utilized in this study. Using this technique, for the first time, we report that NP or SiO_2_ increase lysosomal permeability to cations which could be explained by K^+^ translocating from the cytosol to the lysosomes. Lysosomal K^+^ uptake would explain hyperpolarization of the lysosomal membrane potential leading to osmotic imbalance across the lysosomal membrane and eventually perturbing lysosomal membrane integrity. Furthermore, we speculate that a certain threshold (~ 40 mV) needs to be exceeded in lysosomal membrane hyperpolarization to cause LMP (Figure 8).

Under normal conditions, lysosomes are more acidic and has a much lower [K^+^] than the cytosol, which has high K^+^ levels (140 mM) [23,34]. This high K^+^ gradient is maintained since lysosomes show only a limited permeability toward K^+^ [44]. Furthermore, this regulated lysosomal permeability to cations, with an approximate permeability preference of K^+^>>Na^+^, influences lysosomal pH and protects lysosomes from osmotic lysis [45,46,47]. However, excessive entry and accumulation of K^+^ into lysosomes may contribute to the osmotic disruption of lysosomes [48]. In isolated lysosomes, loss of cholesterol [22], arachidonic acid [21], along with photodamage [20] have been reported to induce an increase in the K^+^-influx, resulting in lysosomal membrane potential alteration and LMP.

The results of the present study showed an increase in lysosomal cation uptake within approximately one hour after exposure of AM to either ZnO or SiO_2_. These findings were inferred from the increase in fluorescent intensity of anionic DiBAC4(3) in the lysosomes of AM incubated with either ZnO or SiO_2_ compared to control cells (Figure 3). Increasing fluorescent intensity of the dye inside the cell has been reported as an indicator of cation uptake [32]. Furthermore, measuring lysosomal membrane potential as described, showed that ZnO or SiO_2_ dose-dependently cause lysosomal hyperpolarization (Figure 4). Since lysosomal hyperpolarization is the result of cation uptake [23], these results provide additional evidence of lysosomal cation uptake attributable to ZnO or SiO_2_ accumulating in phagolysosomes.

Lysosomal hyperpolarization has been reported to be attributable to an influx of two main ions, K^+^ and H^+^, into the lysosomes [23]. However, measuring lysosomal K^+^ in intact cells to confirm K^+^ influx is technically challenging. Therefore, in this study, cytosolic K^+^ was monitored as an indirect procedure to determine the underlying mechanism of lysosomal membrane hyperpolarization. The results are consistent with lysosomal hyperpolarization coincident with decreasing cytosolic K^+^ after exposure of AM to ZnO or SiO_2_ (Figure 5C). In contrast, only a negligible decrease in cytosolic K^+^ was observed in AM treated with TiO_2_ or CeO_2_, which was consistent with the small change in lysosomal hyperpolarization experimental results. Only the high concentration of TiO_2_ (200 µg/mL) simultaneously caused significant cytosolic K^+^ decrease (Figure 5C) and lysosomal hyperpolarization (Figure 4). These findings suggest that lysosomal hyperpolarization likely results from K^+^ influx to lysosomes.

In order to further test the hypothesis that lysosomal hyperpolarization and cytosolic K^+^ decrease in AM treated with ZnO or SiO_2_ was due to translocation to the lysosomes rather than the extracellular space, AM were suspended in K^+^-rich buffer to inhibit K^+^ efflux from the AM. The results showed that ZnO or SiO_2_ still induced lysosomal hyperpolarization (Figure S11) and cytosolic K^+^ decrease (Figure S12), suggesting that lysosomal hyperpolarization and cytosolic K^+^ decrease attributable to ZnO NP or SiO_2_ was likely the result of lysosomal K^+^-influx rather than cytosolic K^+^-efflux. In addition to K^+^-influx, changes in H^+^ inside the lysosome could contribute to a lesser extent to lysosomal membrane hyperpolarization (Figure 6). However, minor acidification of intra-lysosomal pH might be attributable to a high buffering capacity and/or large surface/volume ratio in the lysosome that would cause a more dramatic change in the lysosomal ionic composition upon opening or closing ion transporters.

These studies suggest that in order for any particle to induce sufficient damage to the lysosomal membrane that a certain threshold (~ 40 mV) of lysosomal hyperpolarization needs to occur. Particle-induced lysosomal hyperpolarization exceeding the threshold (Figure 4), resulted in LMP (Figure 7A); furthermore, the titration of Valinomycin indicated that exceeding the threshold (Figure S6A) contributes to LMP (Figure 7D). Moreover, the results indicated that lysosomal membrane potential hyperpolarization is an early event preceding LMP and NLRP3 inflammasome activation. The time-course experiments in AM treated with ZnO or SiO_2_ revealed released cathepsin B in the cytosol, but not earlier than 4 h (Figure 7A–C and Figure S8), while hyperpolarization was detected within 1 h (Figure 4) after incubation of AM with ZnO or SiO_2_. Lysosomal hyperpolarization was also shown to be upstream of NLRP3 inflammasome activity including the release of IL-1β (Figure 7E).

In order to investigate any causal relation between lysosomal membrane potential and LMP, lysosomal membrane hyperpolarization was inhibited using Bafilomycin A1 and its impact on LMP and NLRP3 inflammasome activity was evaluated. Bafilomycin A1 could inhibit lysosomal hyperpolarization (Figure S6A) by increasing the lysosomal pH (Figure S6B). A cocktail of Bafilomycin A1 and ZnO, which slightly hyperpolarized the lysosomal membrane potential (Figure S6A), did not result in LMP (Figure 7D) or NLRP3 inflammasome activity (data not shown). In comparison, ZnO or SiO_2_ alone was able to significantly hyperpolarize the lysosomal membranes (Figure 4), cause significant LMP (Figure 7A), and result in significant release of IL-1β (Figure 7E). These results suggest a causal relation between lysosomal hyperpolarization and LMP/NLRP3 inflammasome activity. In an earlier study, it was suggested that Bafilomycin A1 inhibits LMP by increasing lysosomal pH [28]. This study provided additional evidence indicating the importance of lysosomal ions (K^+^ and H^+^) balance on lysosomal membrane integrity. We suggest that ZnO- or SiO_2_-induced lysosomal hyperpolarization and LMP should be considered as a potential step leading to LMP as illustrated in Figure 8.

Taken together, the findings from the current study provide a plausible explanation to connect a number of findings to explain the activation of the NRLP3 inflammasome and inflammation by particles (both nano-sized particles and crystalline SiO_2_). Cytosolic potassium depletion has emerged as a common denominator in the activation of NLRP3 inflammasome [13,14,15]. Early studies demonstrated that K^+^ ionophores such as nigericin could trigger IL-1β maturation in macrophages [16]. Additional studies suggested that cytosolic K^+^ depletion by nigericin alone is a minimal common cellular event that is sufficient to activate the NLRP3 inflammasome [14,17]. While the findings using nigericin had implicated that depletion of cytosolic K^+^ could be important in NLRP3 inflammasome assembly, it did not explain how a physiologically relevant process (e.g., inhaled inflammatory particles) could accomplish the same changes in cytosolic K^+^.

What remains to be established are to determine the events leading to the flux of K^+^ from the cytosol to the phagolysosome. We recently reported that NP can alter the packing of membrane lipids affecting lipid order [49]. The ability of NP to modify lipid order could lead to the opening of K^+^ channels such as the recently described TEMEM175, a lysosomal K^+^-selective channel [47] or the BK channel. Alternatively, particle-induced disruption of membranes could cause leakage of cations down their concentration gradients. These studies will hopefully stimulate future research into the potential mechanism of cation influx and establish the stepwise sequence of events that may also help in identifying additional pathways to block this pathway leading to particle-induced inflammation.

## 4. Materials and Methods

### 4.1. Reagents

Phorbol 12-myristate, 13-acetate (PMA) was purchased from Sigma-Aldrich (St. Louis, MO, USA) and 1,25-dihydrixy-vitamin D3 from EMD Millipore (Billerica, MA, USA). The cytotoxicity assays CellTiter 96 (MTS assay) and CytoTox 96 [LDH (lactate dehydrogenase) assay] were purchased from Promega (Madison, WI, USA). Amphotericin B was purchased from Cayman Chemical Company (Ann Arbor, MI, USA). ION Potassium Green-2 AM, a K^+^ indicator (Ex/Em 526/546), was purchased from Abcam (Cambridge, UK). LysoTracker Red DND-99 (Ex/Em 577/590), Lysosensor Yellow/Blue DND-160 (Ex/Em 329 and 380/440 and 540), and RPMI 1640 without phenol red were purchased from Thermo Forma/Fisher (Bothell, WA, USA). Gramicidin was purchased from Santa Cruz Biotechnology, Inc. (Dallas, TX, USA). Bafilomycin and bis-(1,3-dibutylbarbituric acid) trimethine oxonol (DiBAC4(3), Ex/Em 493/516) were purchased from Enzo Life Sciences, Inc. (Farmingdale, NY, USA). L-∝-phosphatidylcholine (Egg-PC) and L-∝-phosphatidylethanolamine-N-lissamine rhodamine B sulfonyl (Rh-PE) were purchased from Avanti Polar Lipids (Alabaster, AL, USA).

### 4.2. Particles

ZnO was obtained from Meliorum Technologies Inc. (Rochester, NY, USA). SiO_2_ (Min-U-Sil5) was obtained from Pennsylvania Glass Sand Corp (Pittsburgh, PA, USA) acid washed and dried at 110°C prior to use. CeO_2_ was obtained from Sigma (Cat. #544841; St. Louis, MO, USA). TiO_2_, which was used in prior studies [5], was purchased from Evonik (Parsippany, NJ, USA).

### 4.3. Preparation of NP and SiO_2_ in Cell Culture Media

Stock solutions of NP or SiO_2_ (4 mg/mL) were prepared from dry powder using phosphate buffered saline (PBS pH 7.4) and then all suspensions (0–200 µg/mL) were prepared in RPMI media containing 10% FBS (fatal bovine serum) to be consistent with physiologically relevant matrices containing proteins. The stock solutions were vortexed and then sonicated (550 watts @ 20 kHz) for 2 min in using a water bath sonicator immediately before diluting the solutions into RPMI media.

### 4.4. Mice

Male and female C57B1/6 mice were used in equal numbers for all studies. Animals were housed in micro-isolators in a specific pathogen-free facility under a 12:12-h light-dark cycle. Mice were used between 8 and 12 weeks of age. The University of Montana Institutional Animal Care and Use Committee (Missoula, MT, USA) approved all procedures performed on the animals.

### 4.5. Alveolar Macrophages

AM were isolated from euthanized adult C57BL/6 mice by means of lung lavage using 1.0 mL sterile PBS for four consecutive times in a similar manner as previously described [50]. The isolated cells (>95% AM) were then washed in PBS and resuspended in RPMI 1640 medium supplemented with 10% FBS, 1 mM l-glutamine, and 100 U/mL penicillin-streptomycin (Hamilton et al., 2012). AM were plated in a 96-well plate (1 × 10^5^ cells/well) and exposed to SiO_2_ or NP (0–200 µg/mL) and LPS (20ng/mL) for inflammasome priming. AM were treated with/without Bafilomycin A1 100 nM or Valinomycin (100 nm, 2 µM, and 20 µM), depending on the goal of study and cell supernatants were collected after 1 h. Cellular viability was determined using standard MTS and LDH assays in different time points (1, 2, and 4 h). Cell supernatants were assessed for IL-1β by ELISA (R&D System, Minneapolis, MN, USA).

### 4.6. Preparation of Small Unilamellar Vesicles

Small unilamellar vesicles (SUV) composed of L-∝-phosphatidylcholine (Egg-PC) were prepared by the extrusion method [51]. Egg-PC stock was transferred into a clean 20-mL glass tube using Hamilton syringe and the chloroform was evaporated under a gentle stream of nitrogen at room temperature. Dried Egg-PC was dissolved in a small volume of ethanol and then PBS was added. The solution was vortexed to re-dissolve as much lipid material as possible then sonicated for 10 min at 80 W. The extruder was assembled with filter supports (4 total) and polycarbonate membranes (100 nm, 2 total). The assembled extruder was rinsed with Millipore water three times to remove any air bubbles. The lipid solution was passed ~20 times through the polycarbonate membranes. The SUVs were then exposed to PBS containing 300 nM bis-(1,3-dibutylbarbituric acid) trimethine oxonol DiBAC4(3) for 10 min at room temperature. The buffer was then exchanged with PBS and the fluorescent intensity of the control compared to treatment group was compared with fluorometer.

### 4.7. Lysosomal Membrane Potential Measurement

AM (~1.5 × 10^5^) were first incubated in 35-mm cell culture dishes containing RPMI 1640 medium without phenol red and supplemented with 10% FBS, 1 mM l-glutamine, and 100 U/mL penicillin-streptomycin for 1 h at 37 °C SiO_2_ or NP (0–200 µg/mL) were then added and the cells were incubated in RPMI containing 10% FBS at 37 °C for 4 h. The control groups were incubated in the same experimental condition without NP exposure. The media was then replaced with PBS containing 300 nM DiBAC4(3) and incubated for 10 min at 37 °C. The buffer containing dye was replaced with fresh PBS before acquiring images using a Zeiss LSM 880 confocal microscope. Cells were imaged with a Zeiss 880 laser scanning confocal microscope using a 1.4 N.A. 63× oil immersion objective (Zeiss, San Diego, CA, USA). Emission light was collected by the same objective and delivered to a high sensitivity GaAsP photodetector (Zeiss 880 built-in). All images were acquired with the same excitation laser intensity, pixel dwell time, and detector gain for comparison. The fluorescent intensity of fluorophores was monitored throughout the entire experimental time, and no detectable intensity changes were noted. Brightness and contrast were also set equally for all images.

To measure lysosomal membrane potential, cytosolic to C_C_/C_L_ ratio of DiBAC4(3) was calculated directly based on the fluorescent intensity of DiBAC4(3) in both cytosol and lysosome. To calculate the ratio correctly, the fluorescent intensity of the dye in at least 100 cells per each group was measured and analyzed using ZEN imaging software (Zen 2.3 SP1 FP3 (black) Version 14.0.21.20, from Zeiss) and ImageJ with individual cell approach. Next, the ratio was applied in the equation below to calculate lysosomal membrane potential, where R, T, F, and z are gas constant (8.314 JK^−1^mol^−1^), temperature in Kelvin (301.15 K), Faraday’s constant (96,485.332 Cmol^−1^) and the charge of the DiBAC4(3), respectively [24].

$$\mathrm{Lysosomal}\;\mathrm{membrane}\;\mathrm{potential}\;(\psi \varphi )\;=\;\mathrm{RT}\;{\left(\mathrm{zF}\right)}^{-1}\;\ln\;C_{C}\;{\left(C_{L}\right)}^{-1}$$

Co-localization of DiBAC4(3) and Lysotracker Red in the (phago)lysosomes was qualitatively visualized using confocal microscopy in most cells using a Zeiss LSM 880. Furthermore, in order to remove the bias of visual interpretation, co-localization of the two dyes was quantitatively analyzed in approximately 100 cells using ImageJ and Pearson’s Coefficient was calculated as 0.87 ± 0.08. This coefficient is a well-established measure of co-localization or correlation based on the pixel value and has a range of +1 for perfect correlation, 0 for no correlation, and −1 for perfect anti-correlation [52]. The pH-independency of DiBAC4(3) quantum yield was investigated using a Spectramax M4 fluorescence plate reader. The spectral fluorescent intensity of DiBAC4(3) in PBS at different pHs (i.e., 4.5, 5, 5.5, 6, 6.5, 7, and 7.4) was measured. A calibration curve was generated using increasing concentrations of DiBAC4(3).

### 4.8. Plasma Membrane Potential Measurement

DiBAC4(3) was used in a similar manner as described earlier to measure plasma membrane potential (Warren and Payne, 2015). Cells were first incubated with NP or SiO_2_ (0–200 µg/mL) in RPMI media containing 10% FBS for 1 h. Measurements were conducted by gently removing the semi-adherent AM from the surface of the culture dishes using a rubber scraper, pelleting, and then resuspending the cells in PBS. The suspension of control cells was split into eight 1-mL aliquots in Eppendorf tubes. Tubes were pelleted again, and half of them were resuspended in PBS; the other half were suspended in K^+^-rich buffer containing 135 mM KCl, 5 mM MgCl_2_, 0.5 mM CaCl_2_, 1 mM EGTA, and 10 mM HEPES or PBS with 50 µM amphotericin B to induce depolarization. Both treatment groups and control cells were incubated with 300 nM DiBAC4(3) at 37 °C for 10 min. The buffer was replaced with fresh PBS before monitoring the DiBAC4(3) fluorescent intensity with an Attune NxT flow cytometer. DiBAC4(3) was detected using a FL-1 filter (533/30 BP). For each experiment, ~20,000 cells in the population of interest were sampled. The percent change between the mean intensity of DiBAC4(3) of quadruplicate control samples and depolarized cells was used to measure relative shifts in membrane polarization.

### 4.9. Cytosolic [K^+^] Measurement

APG-2, a cell-permeable K^+^ indicator (Ex/Em 526/546) was used to monitor cytosolic K^+^ in a similar manner as previously described [37,38]. The dye has been previously used as a noninvasive and reliable tool to monitor and quantify cytosolic [K^+^] [37]. Inside the cell, non-specific esterases act to form the active dye. Therefore, the dye specifically monitors cytosolic K^+^. Fluorometric analysis was performed in quartz cuvettes using a SpectraMax M4 spectrofluorometer. The K^+^ selectivity of APG-2 over Na^+^ was analyzed using solutions [37] containing 135 mM KCl, 5 mM MgCl_2_, 0.5 mM CaCl_2_, 1 mM EGTA, and 10 mM HEPES adjusted to pH 7.2, as well as increasing amounts of NaCl. For each K^+^ concentration, emission spectra were recorded.

Intracellular-like solutions were used to develop an in situ calibration curve for K^+^ [37] and contained 12 mM NaCl, 5 mM MgCl_2_, 0.5 mM CaCl_2_, 1 mM EGTA, and 10 mM HEPES adjusted to pH 7.2. AM were permeabilized to monovalent cations using 50 µM amphotericin B and simultaneous inhibition of the Na^+^/K^+^-ATPase using 1 mM Resibufoginin. The K^+^ titration of the dye was obtained by successive additions of known amounts of KCl [37,38] to raise cytosolic [K^+^] to various levels (5 to 145 mM). Cytosolic [K^+^] was then monitored using a SpectraMax M4 spectrofluorometer (San Jose, CA, USA) by loading AM at 37 ℃ for 40 min with 12 µM APG-2. AM (~0.75 × 10^5^) suspended in PBS in 96-well dark plates incubated with NP or SiO_2_ for 1 h at 37 °C. Control AM were incubated in the same buffer for 1 h at 37 °C. The values of cytosolic [K^+^] in control and particle-treated AM were estimated by extrapolating the fluorescent intensity of the dye in the calibration curve. Further experiments were conducted to investigate whether lysosomal K^+^-influx leads to lysosomal hyperpolarization. For this purpose, K^+^-efflux from the cell was inhibited by suspending an equal number of AM in K^+^-rich buffer [37,38]. Given the potential toxic effects of very high extracellular K^+^, the incubation time in K^+^-rich buffer did not exceed 1 h.

### 4.10. Measurement of Lysosomal H^+^

The intralysosomal pH of AM was estimated using ratiometric Lysosensor Yellow/Blue DND-160, which provides for an estimate of lysosomal pH (Ma et al., 2017). The fluorescent intensity of the dye was monitored using a SpectraMax M4 spectrofluorometer. For making a pH calibration curve, nine calibration buffers were made containing 125 mM KCl, 25 mM NaCl, 0.01 mM Monensin, and 25 mM HEPES or 25 mM MES; calibration buffer pHs were adjusted to 3.5, 4, 4.5, 5, 5.5, 6, 6.5, 7, or 7.5 with 1 N HCL/1 N NaOH. AM were seeded onto a 96-well plate at a density of ~3 × 10^4^ cells/well and were incubated at 37 °C in 1 mL pre-warmed RPMI 1640 supplemented with 10% FBS, 1 mM l-glutamine, and 100 U/mL penicillin-streptomycin containing 1 µM LysoSensor Yellow/Blue DND-160 for 5 min. AM were then rinsed with pre-warmed PBS and incubated in 100 µL of their respective pH calibration curve buffers (in triplicate) for 10 min at 37 ℃. The ratiometric measurement of intralysosomal pH was performed using a dual-wavelength fluorescence-based analysis (Ex/Em 329/440 and 380/540), i.e., calculating the ratio of blue fluorescence (in neutral pH) to yellow fluorescence (in acidic pH). The fluorescence intensity ratio was calculated and plotted for each pH calibration curve buffer. To measure intralysosomal pH, cells were seeded on 96-well plates and incubated with particles at a concentration of 0–200 µg/mL for 1 h at 37 °C. Cells were then pelleted and resuspended with 1 mM RPMI containing 1 µM dye under the same incubation conditions as were used to make the calibration curve. AM fluorescence data were collected and the ratio of blue to yellow fluorescence was calculated as previously mentioned for the calibration curve.

### 4.11. Lysosomal Membrane Permeabilization Assay

LMP was assessed as described previously [28]. Briefly, AM were seeded on 96-well plates at a density of 1 × 10^5^ cells per well and incubated at 37 ℃ for 4 h. Cells were then washed with PBS and incubated with 100 µL cytosol extraction buffer plus digitonin. The concentration of digitonin for optimal extraction of the cytosolic fraction was determined by titration. A 1:1 extracted cytosol and cathepsin reaction buffer was prepared, and the fluorescent intensity was read (ex/em: 400/489 nm) using a plate reader for 25 min with 45 s intervals. LDH activity was assessed following manufacturer’s instructions (Promega). Extracted cytosolic LDH activity was used as an internal control to which cytosolic cathepsin B was normalized. Cytosolic extract enzyme activities were calculated as a percent of total cell lysate activity in which 200 µg/mL digitonin was used to completely lyse the cells. The LMP assay was performed using AM incubated with 0–200 µg/mL of SiO_2_ or NP at four different time points (0, 1, 2, and 4 h).

### 4.12. Transmission Electron Microscopy Imaging

NP were tracked inside the AM after a 4 h incubation at 37 °C. Cells were prepared as described earlier [53]. Briefly, AM were washed in clean PBS buffer and fixed with a glutaraldehyde fixative in cacodylate buffer (pH 7.2). The AM were then placed in osmium tetroxide stain and embedded in a small block of 2% agarose. Cells were dehydrated in an ethanol series with 50%, 70%, 90%, 95%, and 2× 100% ETOH. After dehydration, cells were placed in propylene oxide (PO) 1:1 PO and epoxy, 1:2 PO and epoxy, or 100% epoxy. Samples were then ready for microtome sectioning and microscopy. An average particle size was assessed using ImageJ software.

### 4.13. Statistics

Means were compared by *t*-tests using a two-tailed distribution with two-sample unequal variances in PRISM Version 9 (GraphPad, San Diego, CA, USA) software to compare treatment samples to untreated controls for AM. ANOVA statistical analysis and Holm–Sidak’s multiple comparison test were performed using PRISM software to compare differences in means with more than two groups in the experimental design, with *p* ≥ 0.05 being not significant (NS). In contrast, asterisks * *p* ≤ 0.05, ** *p* ≤ 0.01, *** *p* ≤ 0.001, **** *p* ≤ 0.0001 indicated significant effects. Data are presented as means ± SEM of triplicate measurements.

## Data Availability

Not applicable.

## Supplementary Materials

The following are available online at https://www.mdpi.com/1422-0067/22/5/2277/s1.
