# Supplementary material for: Contribution of Particle-Induced Lysosomal Membrane Hyperpolarization to Lysosomal Membrane Permeabilization
Source: Int J Mol Sci. 2021 Feb 25;22(5):2277. doi: 10.3390/ijms22052277 (PMC7956429; doi:10.3390/ijms22052277)
Supplement: Supplementary file 1 [file ijms-22-02277-s001.pdf]

Contribution of particle-induced lysosomal membrane hyperpolarization to lysosomal membrane permeabilization

Tahereh Ziglari[1], Zifan Wang[2], Andrij Holian[1]*

Supplementary data

Table S1. Physiochemical characteristics of ENM and SiO_2.

Quality	Technique	ZnO NP	TiO_2	CeO_2	SiO_2
Size (nm)	TEM	~30	~25	~25	500-1000
Size in RPMI media (nm ±SD)	DLS	215±15	1025±137	1154±140	1029±165
Zeta potential in H_2O at pH 6 (mV±SD)	Zetasizer	-28.2±0.5	17.3±0.9	24.8±0.49	-46±1.48

Table S1. Physiochemical characteristics of three NP and SiO_2. Average particle size was assessed using ImageJ software. DLS measurement was based on intensity.

Table S2. Lysosomal membrane potential calculation using Nernst equation.

	C_C/C_L ratio of ~100 cells per group	Lysosomal membrane potential ($\psi\phi$)= RT/zF ln C_C/C_L
Control	58.27/142.89 = 0.4	0.023 J/C = 0.023 V= 23 mV
ZnO	60.43/600.12= 0.1	0.06 J/C = 0.06 V= 60 mV
TiO_2	62.76/223.45= 0.223	0.04 J/C = 0.04 V= 40 mV
CeO_2	54.32/234.78= 0.23	0.038 J/C = 0.038 V= 38 mV
SiO_2	56.24/466.66= 0.12	0.055 J/C = 0.055 V= 55 mV

Table S2. Lysosomal membrane potential calculation using Nernst equation. AM were incubated with individual NP or SiO_2 for 1 hr at 37°C. C_C/C_L ratio was measured in 100 cells per each group and lysosomal membrane potential was calculated as discussed in the Methods.

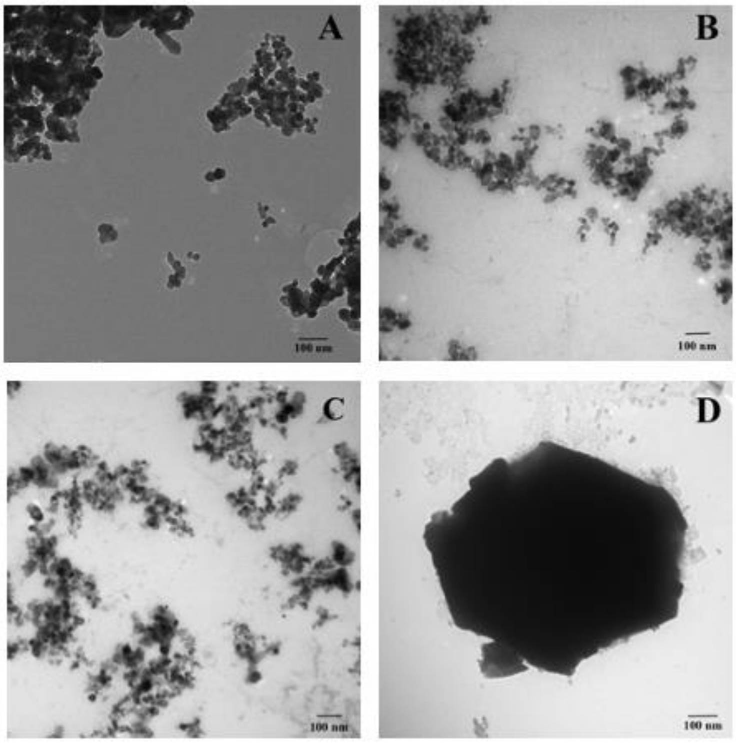

Figure S1. Transmission electron microscopy images of NP and SiO_2 in PBS buffer. **A)** ZnO, **B)** TiO_2, **C)** CeO_2, and **D)** SiO_2 are shown. An average particle size was assessed using ImageJ software.

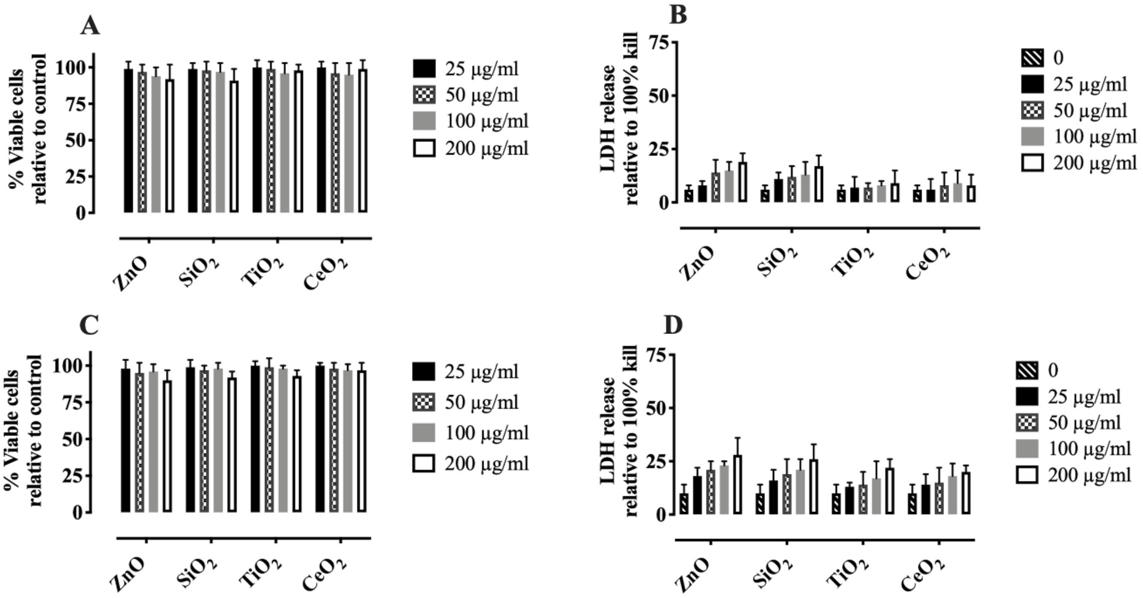

Figure S2. Time-course toxicity in AM attributable to NP and SiO_2. AM were incubated with individual particles for 1 and 2 hr at 37°C. Results from the **A)** MTS assay after incubation of particles with AM for 1 hr, **B)** LDH assay after incubation of particles with AM for 1 hr, **C)** MTS assay after incubation of particles with AM for 2 hr **D)** LDH assay after incubation of particles with AM for 1 hr. Data are presented as means ± SE of triplicate measurements.

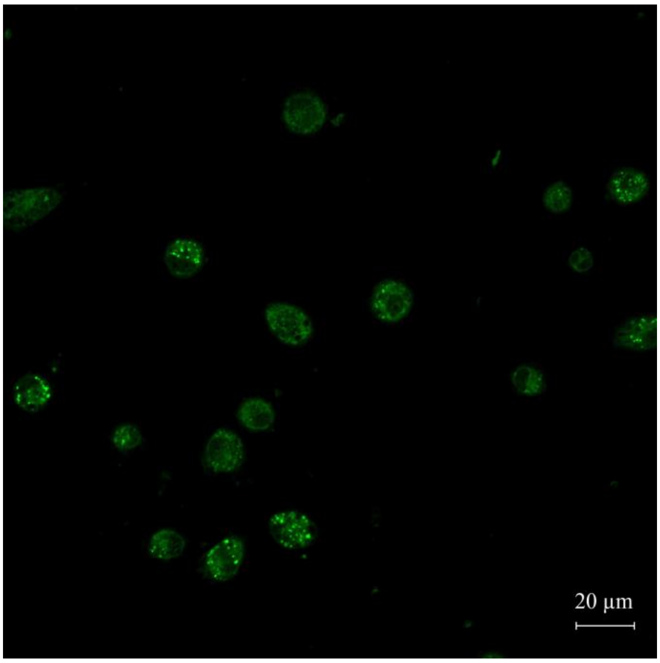

Figure S3. Accumulation of DiBAC4(3) in the lysosomes of AM. AM were incubated with 300 nm DiBAC4(3) for 10 min in PBS at 37°C. A Zeiss LSM 880 confocal microscope was used to acquire the image.

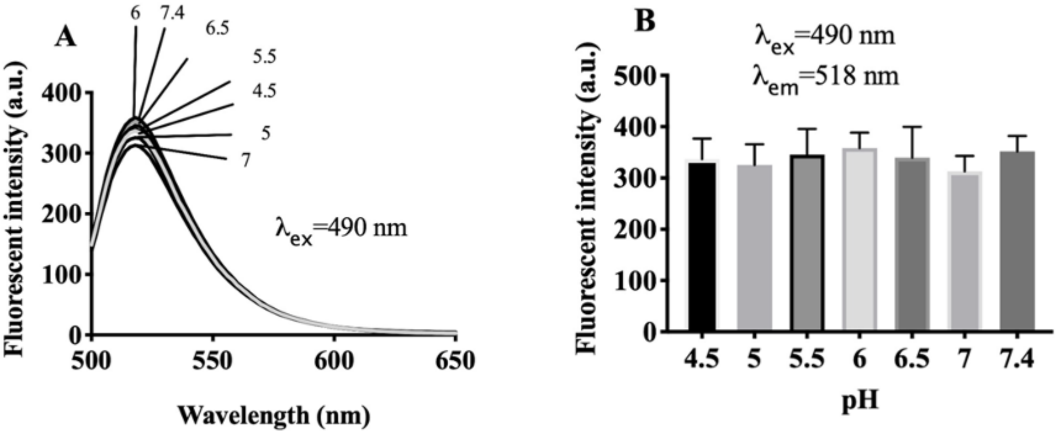

Figure S4. Lack of pH dependency of the DiBAC4(3) quantum yield. **A)** Spectra of fluorescent intensity of DiBAC4(3) in PBS at different pHs. Fluorimetric analysis was performed in quartz cuvettes using a Spectramax M4 fluorescence spectrometer. **B**) Fluorescent intensity of DiBAC4(3) in PBS at different pHs (emission 518 nm). Data are presented as means ± SE of triplicate measurements.

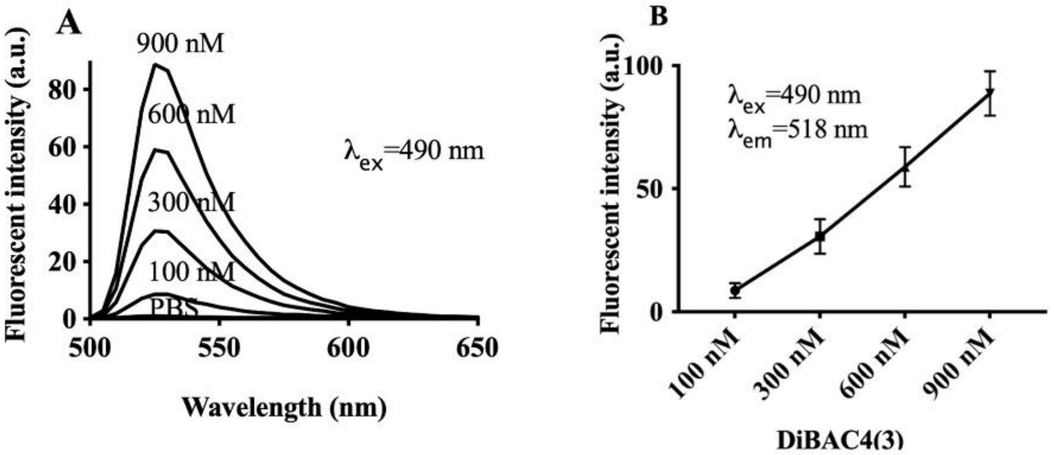

Figure S5. Fluorescence intensity of DiBAC4(3) at different concentrations. **A)** Fluorescence emission traces of a titration of DiBAC4(3). Fluorimetric analysis was performed in quartz cuvettes using a Spectramax M4 fluorescence spectrometer. **B)** The calibration curve was calculated based on the peak fluorescent intensity of each concentration at 518 nm.

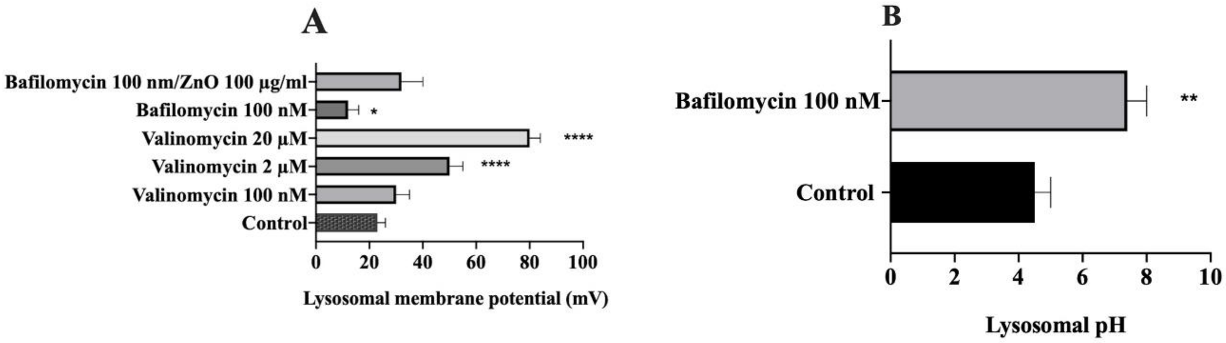

Figure S6. The importance of K^+ and H^+ in maintaining lysosomal membrane potential. A) Lysosomal membrane potential was manipulated with Valinomycin or Bafilomycin A1. AM were incubated with Valinomycin or Bafilomycin A1 for 1 hr at 37°C and lysosomal membrane potential was determined as discussed in the Methods. **B)** The effect of Bafilomycin on lysosomal pH. AM were incubated with Bafilomycin A1 for 1 hr at 37°C and lysosomal pH was determined with LysoSensor Yellow/Green as discussed in the Methods. Data are presented as means ± SE of triplicate measurements. *, **, and **** indicates significant effect (P≤0.05, P≤0.01, and P≤0.0001, respectively).

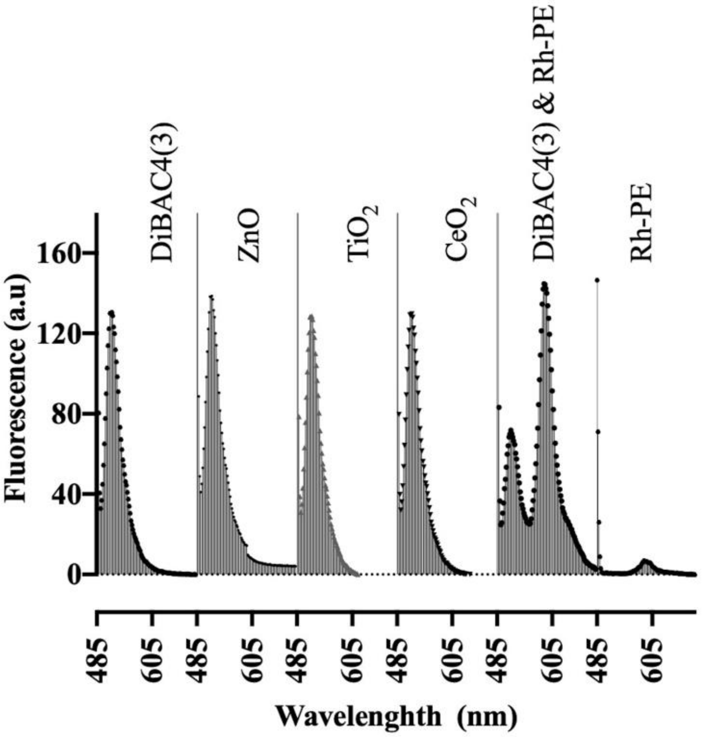

Figure S7. NP do not interfere with DiBAC4(3) or the fluorescent signal generated. Small unilamellar vesicles composed of L-∝-phosphatidylcholine (Egg-PC) with or without individual NP were prepared by the extrusion method. The emission fluorescent spectra of liposome with and without NP was measured with spectrophotometry as discussed in the Methods. Liposome containing DiBAC4(3) and its quencher, L-∝-phosphatidylethanolamine-N-lissamine rhodamine B sulfonyl (Rh-PE) was used as a control for fluorescent intensity reduction of DiBAC4(3). The area under curve of the spectra (liposome with and without NP) was calculated using Prism software.

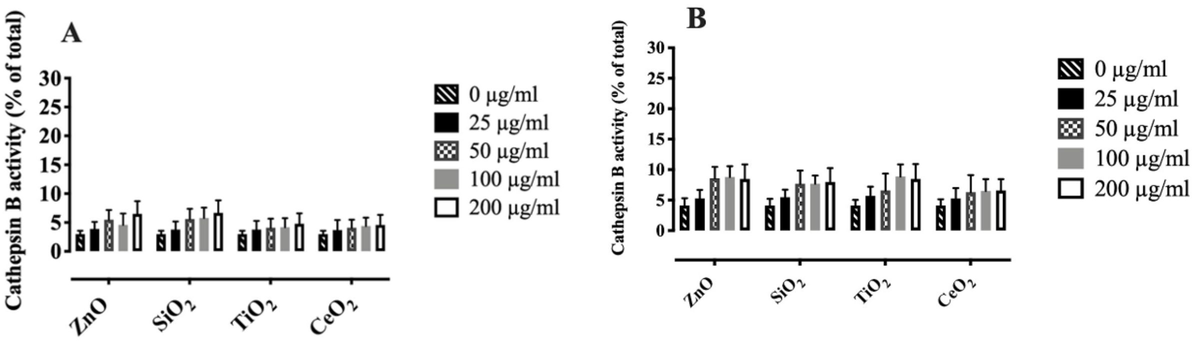

Figure S8. Lysosomal membrane permeabilization of AM treated with NP or SiO_2. AM were incubated with individual particles for 1 and 2 hr at 37°C and cathepsin B release was determines as discussed in the Methods. Results from the release of cathepsin B into the cytoplasm was used as an indicator of LMP and was evaluated

using digitonin as described in Methods. LMP assay after incubation of particles with AM for **A)** 1 hr, **B)** 2 hr. Data are presented as means ± SE of triplicate measurements.

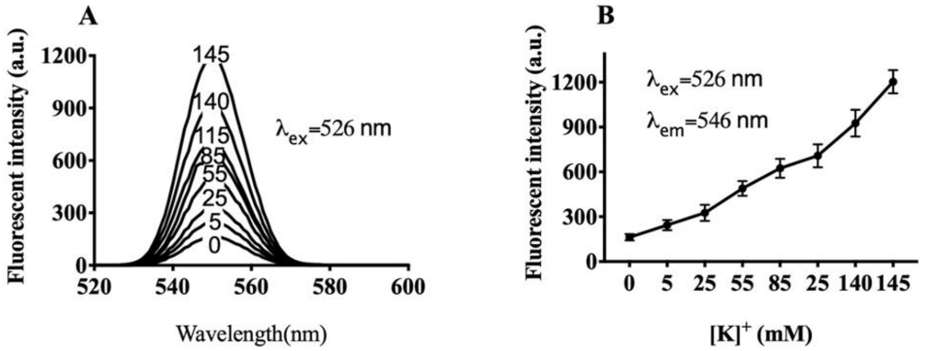

Figure S9. Spectrofluorimetric characterization of APG-2, a K^+ indicator. A) Emission spectra recorded in the presence of different K^+ in intracellular-like solutions as described in Methods. Fluorimetric analyses were performed in quartz cuvettes using a Spectramax M4 fluorescence spectrometer. **B)** Fluorescence emission plotted as a function of K^+ showing a positive relationship of K^+ indicator fluorescence with increasing K^+.

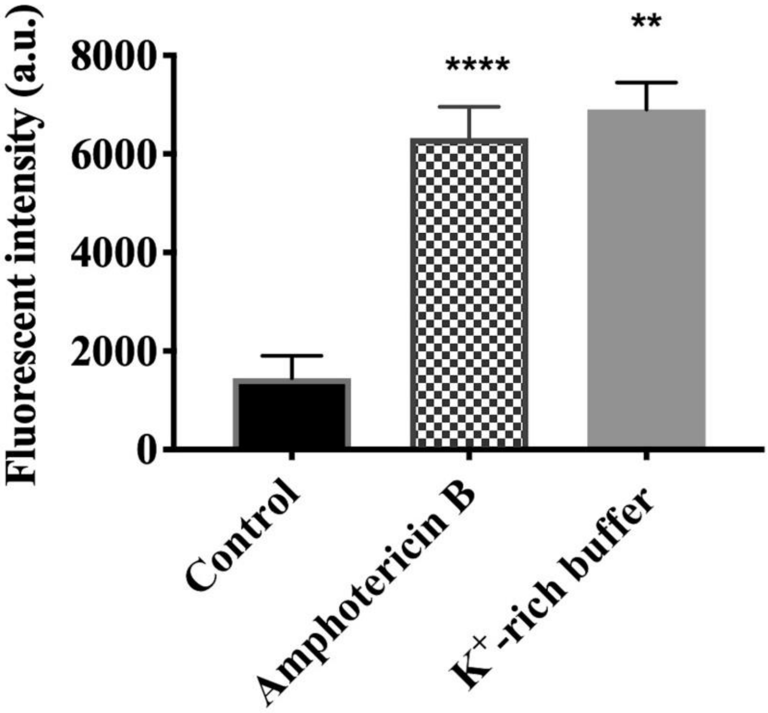

Figure S10. Amphotericin B-, or K^+-rich buffer-induced plasma membrane depolarization in alveolar macrophages (AM). Plasma membrane potential was

measured as described in Methods. Data are presented as means ± SE of triplicate measurements. **** Indicates significant effect (P≤0.0001).

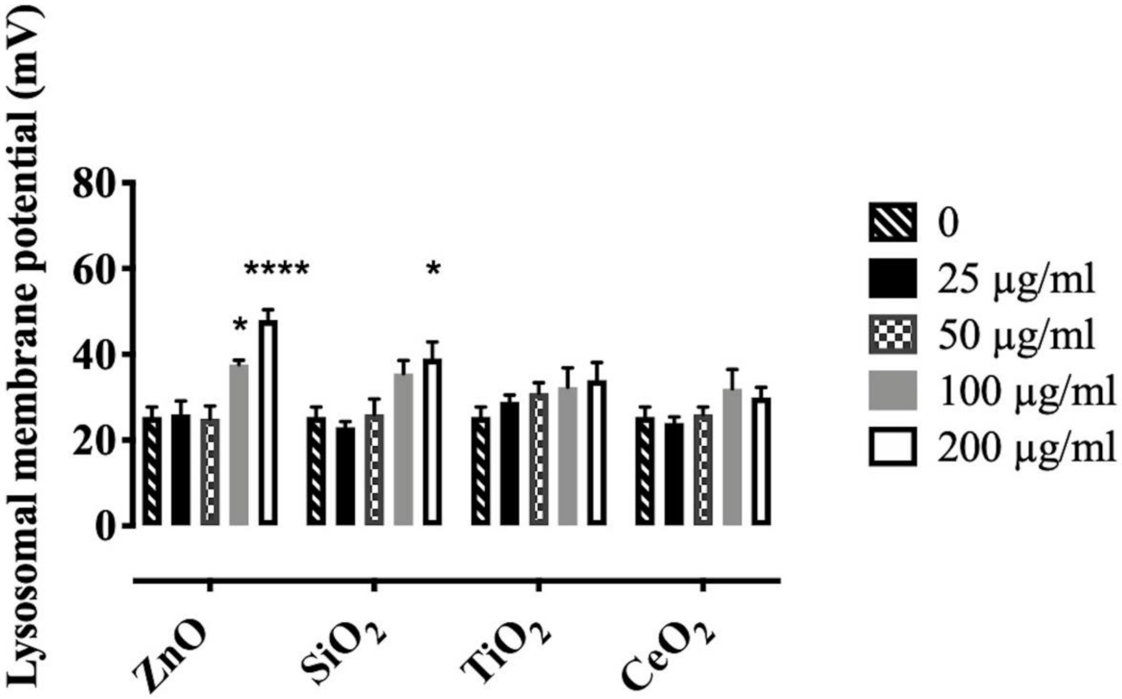

Figure S11. Particle-induced hyperpolarization is the result of lysosomal K^+ influx rather than cytosolic K^+ efflux. AM suspended in K^+-rich buffer were incubated with individual particles for 1 hr at 37℃ . Control cells were incubated in the same experimental condition without particle. Lysosomal membrane potential changes (hyperpolarization) were calculated as described in Methods. A Zeiss LSM 880 confocal microscope and ZEN imaging software (ZEISS) as well as ImageJ were used for our studies. The fluorescent intensity of at least 100 cells was measured and analyzed using ZEN imaging software (ZEISS) and ImageJ. Statistical analysis was performed using Prism software. Data are presented as means ± SE of triplicate measurements. *, **, and **** indicate significant effects (P≤0.05, P≤0.01 and P≤0.0001, respectively).

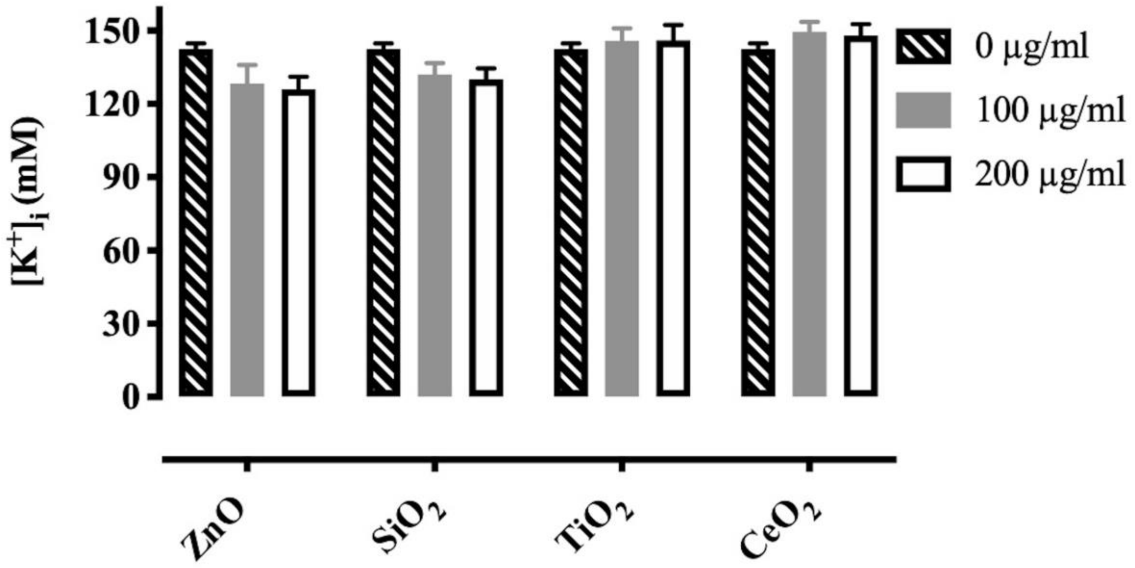

Figure S12. Cytosolic K^+ decrease due to particles is the result of lysosomal K^+ influx rather than cytosolic K^+ efflux. Cytosolic K^+ was measured using acetoxymethyl ester of APG-2 and a SpectraMax M4 spectrofluorometer as described in Methods. AM suspended in K^+-rich buffer were incubated with NP or SiO_2 for 1 hr at 37°C. Data are presented as means ± SE of triplicate measurements.

1.
